# Ohio State Dental Society

**Published:** 1885-12

**Authors:** 


### EDITORIAL.

### Ohio State Dental Society.

The first annual meeting of the Ohio State Dental Society since its reorganization was held in Chillicothe, October 28th, 29th, and 30th. This, to say the least, was a very encouraging meeting, especially in view of the fact that there had been some doubt in the minds of many as to the results that would be accomplished; there was, however, quite a large number in attendance and a good degree of enthusiasm manifested. There seemed to be a determination with every one that the society under its new auspices should be successful, and that it should equal, if not exceed, its former prosperity. The organization was effected in a very thorough manner; a constitution and by-laws framed and adopted, based upon past experience, not only in this State, but in other States as well. The organization occupied nearly two whole days, such was the careful consideration bestowed upon every point; the utmost harmony prevailed, and it is to be hoped that in the future discord will never enter.

The matter of the amendment, or revision of the law regulating the practice of dentistry in this State, received much careful thought and consideration, and arrangements were made for securing the enactment of such a law. Quite a number of papers were read and discussions had upon them, which gave evidence of progress in the science of our profession, and though comparatively short time was devoted to this part of the work, yet many new and instructive ideas were brought out. The arrangements for the meeting and the accommodations for the members were so good, that there was a strong inclination to have the meeting of next year held in Chillicothe; the profession there had done nobly in this respect, but it was finally decided to hold the next annual meeting in Toledo, where we hope and believe there will be the largest meeting ever held in the State, and it certainly will be if the interest aroused in Chillicothe is continued for the year to come.

